# The relationship between polymorphism of insulin-like growth factor I gene and susceptibility to type 2 diabetes in Uygur population, Xinjiang, China

**DOI:** 10.1007/s13258-021-01209-6

**Published:** 2022-01-30

**Authors:** Tingting Wang, Gulixiati Maimaitituersun, Haonan Shi, Cheng Chen, Qi Ma, Yinxia Su, Hua Yao, Jia Zhu

**Affiliations:** 1 School of Nursing and Health Management, Shanghai University of Medicine and Health Sciences, Shanghai, 201318 China; 2 School of Public Health, Xinjiang Medical University, Urumqi, 830054 China; 3 Clinical Laboratory Center, People’s Hospital of Xinjiang Uygur Autonomous Region, Urumqi, 830001 China; 4 Xinjiang Key Laboratory of Metabolic Disease, Clinical Medical Research Institute, The First Affiliated Hospital of Xinjiang Medical University, No.137. Liyushan road, Xinshi District, Urumqi, 830001 China; 5 Health Management Institute, Xinjiang Medical University, Urumqi, 830054 China; 6 Cadre Health Center, People’s Hospital of Xinjiang Uygur Autonomous Region, No. 91, Tianchi Road, Tianshan District, Urumqi, 830001 China

**Keywords:** *IGF-1* gene, Gene polymorphism, Type 2 diabetes, Uyghur

## Abstract

**Background:**

Type 2 diabetes (T2DM) susceptibility varies among different populations and is affected by gene single nucleotide polymorphism (SNP). Insulin-like growth factor (*IGF*)-1 gene, which has many SNP loci, is involved in T2DM pathogenesis. However, the relationship of *IGF-1* gene polymorphism with T2DM in Uyghur population is less studied.

**Objective:**

To investigate the relationship between T2DM susceptibility and polymorphism of *IGF-1* gene in Uyghur population of Xinjiang, China.

**Methods:**

This study enrolled 220 cases (122 males (55.46%) and 98 females (44.54%); mean age of 53.40 ± 10.94 years) of T2DM patients (T2DM group) and 229 (124 males (54.15%) and 105 females (45.85%); mean age of 51.64 ± 10.48 years) healthy controls (control group). Biochemical indexes were determined. *IGF-1* gene polymorphism was analyzed by SNP genotyping.

**Results:**

The levels of TG, HDL, LDL, BUN, and Cr were statistically significant between the T2DM group and the control group. In terms of *IGF-1* polymorphism, T2DM group had higher frequency of AA genotype (OR = 2.40, 95% CI = 1.19–4.84) and allele A (OR = 1.55, 95% CI = 1.17–2.06) of rs35767 loci, suggesting that rs35767 is related to the occurrence of T2DM. A total of 5 gene interaction models was obtained through analyzing the interaction of 5 SNP loci with the GMDR method. Among them, the two-factor model that included rs35767 locus and rs5742694 locus had statistical difference with a large cross-validation consistency (10/10). The combination of GG/CC, GA/AA, AA/AA, and AA/AC genotype was in high-risk group, whereas the combination of GG/AA, GG/AC, GA/AC and GA/CC genotype was in the low-risk group. The risk of T2DM in the high-risk group was 2.165 times than that of the low-risk group (OR = 2.165, 95% CI = 1.478–3.171).

**Conclusion:**

TG, HDL, LDL, BUN, and Cr are influencing factors of T2DM in Uyghur population. The rs35767 locus of *IGF-1* gene may be associated with T2DM in Uyghur population. The high-risk group composing of rs35767 locus and rs5742694 locus has a higher risk of T2DM.

## Introduction

Type 2 diabetes (T2DM) is a chronic metabolic disease with abnormally high blood glucose caused by insufficient insulin secretion or insulin resistance (Ahmed et al. 2020). Like other metabolic diseases, T2DM is affected by both genetic and environmental factors, and is related to the occurrence of many diseases (Sarega et al. 2016). The prevalence of T2DM varies among different populations and regions. For example, it has been shown that the prevalence of T2DM in Uyghur population of Xinjiang, China is 8.16%, and that in Kazakh population is 1.47% (Tao et al. 2008). Another survey showed that the prevalence of T2DM in Xinjiang Uygur population was 9.5% (Awuti et al. 2012).

It is known that there are 56 ethnic groups in China. The Han nationality is the dominant ethnic group in China and its population accounts for 91.11% of the total population; while the population of the other 55 ethnic groups accounts for 8.89%. The majority of the Uyghur ethnic group lives in the Xinjiang Uyghur Autonomous Region, mainly in the south of the Tianshan Mountains (including Hotan, Kashgar, and Aksu). According to the seventh national census statistics of China conducted in Xinjiang on 2020, the Uyghur, as the dominant ethnic minority, has a population of 11.6243 million, accounting for 44.96% of the population in Xinjiang, and, the Han ethnic group has a population of 10.9201 million, accounting for 42.24%. The genetic and environmental factors of T2DM of Han and other ethnic groups have been extensively studied (Hu and Jia 2018; Gong et al. 2019). However, such studies on the Uyghur population in Xinjiang are less reported.

Insulin-like growth factor I (IGF-1) regulates cell proliferation and is essential for mammalian growth and development (Kwasniewski et al. 2015). IGF-1 promotes the uptake of glucose by tissues, stimulates gluconeogenesis and glycolysis through insulin-like effects, thereby promoting protein and fat synthesis and playing an important role in cell proliferation and apoptosis (Mancuso et al. 2017; Yakar et al. 2002). Gene polymorphism, especially single nucleotide polymorphism (SNP), is an important factor affecting the susceptibility and phenotype of T2DM (Bakhashab et al. 2020; Kodama et al. 2018). The *IGF-1* gene is located on chromosome 12, with a total length of 84,734 bp, and contains 3103 SNP loci (Franco et al. 2014). These polymorphic sites may have different functions due to their different positions, such as regulating gene expression levels, and affecting gene post-transcriptional regulation or amino acid sequence (Qin et al. 2019). For example, it has been reported that the rs35767 and rs5742612 loci of the *IGF-1* gene are related to coronary artery damage (Fujita et al. 2012). The rs2162679 and rs6214 loci are correlated with diabetic eye diseases (Hu et al. 2010; Xing et al. 2014). However, the relationship of *IGF-1* gene polymorphism with T2DM is less studied.

Herein, this study investigates the relationship between *IGF-1* gene polymorphism and T2DM in the Uyghur population from Urumqi, Xinjiang. The rs5742694, rs5742612, rs6218, rs35749 and rs35767 SNP of *IGF-1* gene was analyzed. Our findings may provide a scientific basis for the prevention and treatment of T2DM.

## Materials and methods

### Subjects

**Fig. 1:**
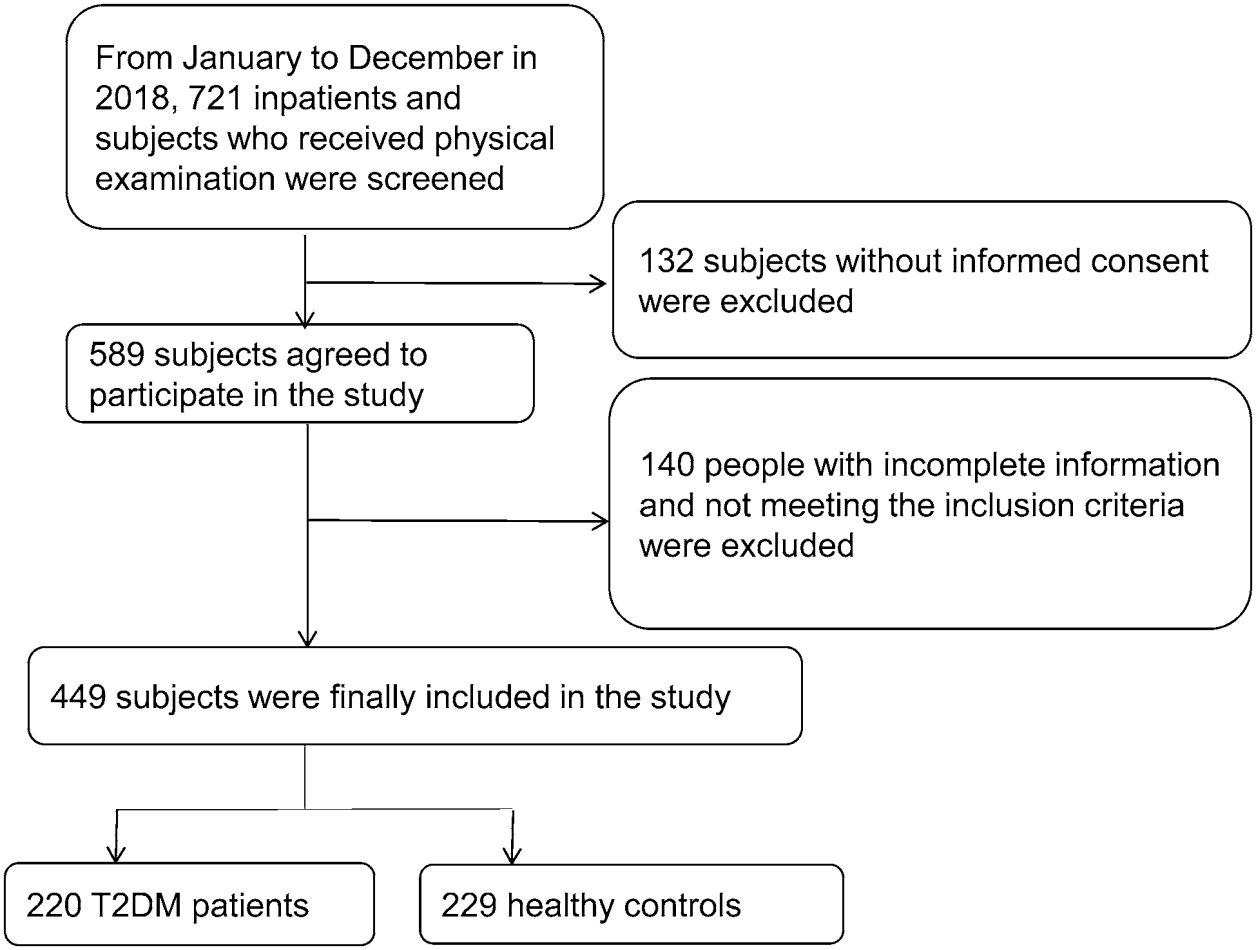
The detailed subject recruitment process

At first, we screened 721 subjects and obtained informed consent from 589 subjects. Among the 589 subjects, 449 patients met the inclusion criteria, including 220 patients with T2DM and 229 control subjects. In detail, the 220 patients with T2DM were hospitalized in the First Affiliated Hospital of Xinjiang Medical University from January to December 2018. They were aged 20–70 years old. Meanwhile, the 229 controls were age-matched healthy subjects who received physical examination during the same period. None of them had hyperglycemia or hyperuricemia. Inclusion criteria: (1) Uyghur patients lived in Urumqi, Xinjiang; (2) Patients with fasting blood glucose ≥ 7.0 mmol/L (Yakar et al. 2002) or those who have been diagnosed with T2DM; Exclusion criteria: (1) patients with blood diseases (such as polycythemia, myelodysplasia, multiple myeloma, acute and chronic hemolytic anemia, leukemia, and lymphoma) and other malignant tumors, were excluded; (2) patients with non-gouty kidney stones, kidney failure and other kidney diseases were excluded; (3) patients who had recently taken medications (such as thiazide diuretics, aspirin, and pyrazinamide) that could induce blood uric acid (UA) increase were excluded. The detailed subject recruitment process is shown in Fig. 1. All subjects signed the informed consent. This study was approved by the Ethics Committee of the First Affiliated Hospital of Xinjiang Medical University.

### Questionnaire survey and physical examination

The structured self-designed questionnaires that combined the living habits and ethnic customs of the Xinjiang Uyghur population were used. Trained investigators proficient in the Uyghur language conducted one-to-one inquiries and physical examinations to obtain basic information. The survey information included disease history, diet and living habits, and family history, etc.; T2DM information (the course of T2DM, diagnosis age, confirmed blood glucose, highest blood glucose, complications and concomitant diseases); risk factors related to UA; lifestyle (physical exercise, smoking, drinking, tea drinking and diet control after illness, etc.); social and psychological factors, etc*.*

### Determination of biochemical indicators

Peripheral venous blood (2 mL) was collected after 12 h of fasting. Serum was isolated within 2 h. The concentration of UA in serum was determined by the uricase method. Serum levels of triglyceride (TG), total cholesterol (TC), high-density lipoprotein (HDL), low-density lipoprotein (LDL), urea nitrogen (BUN), and creatinine (Cr) were all measured on the Abbott ARCHITECT CI16000 biochemical analyzer (Genesky Biotechnologies Inc., Shanghai, 201315).

### Genotype analysis

The DNA was extracted from peripheral blood samples using the DNA extraction kit (QIAGEN, Germany). A multiplex PCR-ligase detection reaction method was used for genotyping of five SNPs. For each SNP, the alleles were distinguished by different fluorescent labels of allele specific oligonucleotide probe pairs. Different SNPs were further distinguished by different extended lengths at the 3’-end. Negative quality control, Hardy–Weinberg Equilibrium and Minor Allele Frequency analysis on typing results were then carried out. The primers used in this study are listed in Table 1.

**Table 1:** Primer information for IGF-1 gene genotyping

SNP	Chromosome position	SNP property	PCR primer	Ligation primer
rs5742694	102,799,236	Intron3	rs5742694F: CCTGGCCGTGTTCTGCTTTC rs5742694FR: TGCGGGTTTGATCAGCTACAGA	rs5742694FA: TACGGTTATTCGGGCTCCTGTCATGGGGCCACAGAATAGCAGCA rs5742694FC: TTCCGCGTTCGGACTGATATCATGGGGCCACAGAATAGCAACC rs5742694FP: CTAATGTTCCCAAACTCCTTGAAATGTTTTTTT
rs5742612	102,874,864	5ʹ-flanking	rs5742612_rs2288377F: GGGGGATGGGAGAGCAATTTTA/rs5742612_rs2288377R: GCCTGCCCCTCCATAGGTTCTA	rs5742612FA: TACGGTTATTCGGGCTCCTGTCCTTGTCCCAGTTGCCAAGTGTGA rs5742612FG: TTCCGCGTTCGGACTGATATCCTTGTCCCAGTTGCCAAGTGTGG rs5742612FP: GGTGTGATCTCATTTCCTAGAACCTATGTTTTTTT rs5742694FA: TACGGTTATTCGGGCTCCTGTCATGGGGCCACAGAATAGCAGCA rs5742694FC: TTCCGCGTTCGGACTGATATCATGGGGCCACAGAATAGCAACC
rs6218	102,793,633	3ʹ-UTR_exon5	rs6218F: TTGGGGGATTTTTGACTGTGGA rs6218R: CCCTCTGCCTGTTTTCCAGACA	rs6218RA: TACGGTTATTCGGGCTCCTGTGGCACTTCTTTTTATTTCTTGTCCCCTGT rs6218RG: TTCCGCGTTCGGACTGATATGGCACTTCTTTTTATTTCTTGTCCCCTGC rs6218RP: GTGTACCTTTTAAAATTATTCCCTCTCAACATT
rs35749	102,940,183	5ʹ-flanking	rs35749F: GGGTTGATATGGGGAGGGTGTT rs35749R: GCCCAATGATATGAAAGCCCAAAT	rs35749RC: TCTCTCGGGTCAATTCGTCCTTCGATACATGGGTTCTTTCAGCAAATTGTG rs35749RP: TCCTATCTTCCTCAGGAACTGATAAAGTTCTTTTTT rs35749RT: TGTTCGTGGGCCGGATTAGTCGATACATGGGTTCTTTCAGCAAATTGTA
rs35767	102,875,569	5ʹ-flanking	rs35767F: GAGCAGACATACCTCTTTCCCTAGAGAGC rs35767R: GATTTCAAGCAGAACTGTGTTTTCAGTTG	rs35767RA: TACGGTTATTCGGGCTCCTGTTGTCAGTCCCCTGAGAGTCACGT rs35767RG: TTCCGCGTTCGGACTGATATTGTCAGTCCCCTGAGAGTCACGC rs35767RP: GGAAAAAACAAAAAAGAAAAAATTCAAGGTCCAGGTTATTTCCAC

### Statistical analysis

SPSS20.0 software was used for statistical analysis. The count data was expressed as percentage, and the measurement data was expressed as mean ± SD. The comparison between the two groups was performed by the Chi-Square (*χ*^2^) test or the rank sum test. The generalized multi-factor dimensionality reduction (GMDR) software was used for the interaction analysis of the SNPs loci of the *IGF-1* gene, and for selecting the optimal gene interaction model. The MDR software was used to plot on-site interactive radiographs to determine whether the interaction is antagonistic or synergistic. *P* ≤ 0.05 indicates that the difference is statistically significant.

## Results

### Basic information of subjects

The basic clinical data of patients is shown in Table 2. There were 220 cases in the T2DM group, including 122 (55.46%) males and 98 (44.54%) females, with an average age of 53.40 ± 10.94 years. There were 229 cases in the control group, including 124 males (54.1%) and 105 females (45.9%), with an average age of 51.64 ± 10.48 years (Table 2). The gender and age of the T2DM group and the control group were not statistically significant (*P* > 0.05). The comparison of urban and rural residents and educational level between the two groups was statistically significant (*P* < 0.05). Importantly, the levels of TG, HDL, LDL, BUN, and Cr were statistically significant between the T2DM group and the control group (*P* < 0.05) (Table 3). There were no significant differences in other factors (*P* > 0.05).

**Table 2:** Basic data of the enrolled subjects

Index	T2DM group (*n* = 220)	Control group (*n* = 229)	*χ*^2^/*t* valu*e*	*P* value
Male	122 (55.46%)	124 (54.15%)	0.106	0.744
Female	98 (44.54%)	105 (45.85%)		
Age	53.40 ± 10.94	51.64 ± 10.48	− 1.739	0.083
Citizens	111 (50.45)	198 (86.46%)	67.80	0.000
Education				
College degree and above	151 (68.63%)	180 (78.60%)	15.75	0.016
Below college	69 (31.37%)	49 (21.40%)

**Table 3:** Comparison of biochemical indicators between the T2DM group and control group (mean ± SD)

Index	T2DM group	Control group	*t/tʹ*	*P* value
BMI (kg/m^2^)	26.50 ± 3.34	26.91 ± 4.09	1.195	0.233
TG (mmol/L)	2.11 ± 1.32	1.76 ± 1.41	− 2.682	0.008
TC (mmol/L)	4.19 ± 1.14	4.02 ± 1.43	− 1.319	0.157
HDL (mmol/L)	1.04 ± 0.56	1.35 ± 0.55	5.840	0.000
LDL (mmol/L)	3.03 ± 1.43	2.54 ± 1.00	− 4.158	0.000
BUN (mmol/L)	4.50 ± 1.81	4.91 ± 1.95	2.261	0.024
Cr (μmol/L)	49.81 ± 24.18	78.06 ± 20.81	13.492	0.000

*BMI* Body Mass Index, *TG* Triglyceride, *TC* Total cholesterol, *HDL* High density lipoprotein, *LDL* Low density lipoprotein, *BUN* Blood urea nitrogen, *Cr* Creatinine

### Hardy–Weinberg equilibrium test of IGF-1 gene polymorphism in the T2DM group and the control group

In this study, the Hardy–Weinberg Equilibrium test was also performed on the 5 loci of the *IGF-1* gene in the T2DM group and the control group. The results showed that the differences of all loci were not statistically significant (*P* > 0.05, Table 4), indicating that these 5 loci have reached a genetic balance in the Uyghur population and have good population representativeness.

**Table 4:** Hardy–Weinberg equilibrium test results

Locus	Genotype	Control group		T2DM group	
		Observed value	Expected value	Observed value	Expected value
rs35749	C/C	189	194.1	579	573.9
	C/T	38	32.9	92	97.1
	T/T	2	2	6	6
	*χ*^2^	0.043		0.121	
	*P*	0.979		0.941	
rs35767	G/G	120	111.7	322	330.3
	G/A	95	96.6	287	285.4
	A/A	14	20.7	68	61.3
	*χ*^2^	0.027		0.053	
	*P*	0.987		0.974	
rs5742612	A/A	167	160	466	473
	G/A	58	62.4	189	184.6
	G/G	4	6.6	22	19.4
	*χ*^2^	0.083		0.145	
	*P*	0.959		0.93	
rs5742694	A/A	138	145.3	437	429.7
	C/A	80	73.3	210	216.7
	C/C	11	10.4	30	30.6
	*χ*^2^	0.021		0.336	
	*P*	0.99		0.845	
rs6218	A/A	175	166.8	49	57.1
	A/G	49	57.1	177	168.9
	G/G	5	5.1	15	14.9
	*χ*^2^	0.038		0.175	
	*P*	0.981		0.916	

### Comparison of SNP sites of IGF-1 gene between the T2DM group and the control group

A comparison of *IGF-1* gene polymorphism between the T2DM group and the control group showed that there were no significant differences in rs35749, rs5742612, rs5742694, and rs6218 loci (*P* > 0.05). Compared with the control group, T2DM group had higher frequency of AA genotype (OR = 2.40, 95% CI = 1.19–4.84) and allele A (OR = 1.55, 95% CI = 1.17–2.06) of rs35767 loci, suggesting that rs35767 is related to the occurrence of T2DM (Table 5).

**Table 5:** The *IGF-1* gene polymorphism in the T2DM group and the control group

SNP	Genotype/allele	Control group *n* (%)	T2DM group *n* (%)	OR (95%CI)	*P* value
rs35749	C/C	189 (82.5)	193 (87.7)	1.000	
	C/T	38 (16.6)	26 (11.8)	0.67 (0.39–1.15)	0.140
	T/T	2 (0.9)	1 (0.5)	0.49 (0.04–5.45)	0.560
	C	416 (90.8)	412 (93.6)	1.00	
	T	42 (9.2)	28 (6.4)	0.67 (0.41–1.11)	0.120
rs35767	G/G	120 (52.4)	93 (42.3)	1.00	
	G/A	95 (41.5)	101 (45.9)	1.37 (0.93–2.03)	0.110
	A/A	14 (6.1)	26 (11.8)	2.40 (1.19–4.84)	0.020
	G	335 (73.1)	287 (65.2)	1.00	
	A	123 (26.9)	153 (34.8)	1.55 (1.17–2.06)	0.000
rs5742612	A/A	167 (72.9)	142 (64.5)	1.00	
	G/A	58 (25.3)	73 (33.2)	1.48 (0.98–2.23)	0.060
	G/G	4 (1.7)	5 (2.3)	1.47 (0.39–5.58)	0.570
	A	392 (85.6)	357 (81.1)	1.00	
	G	66 (14.4)	83 (18.9)	1.38 (0.97–1.97)	0.070
rs5742694	A/A	138 (60.3)	150 (68.2)	1.00	
	C/A	80 (34.9)	59 (26.8)	0.68 (0.45–1.02)	0.060
	C/C	11 (4.8)	11 (5.0)	0.92 (0.39–2.19)	0.850
	A	356 (77.7)	359 (81.6)	1.00	
	C	102 (22.3)	81 (18.4)	0.79 (0.57–1.09)	0.150
rs6218	A/A	175 (76.4)	153 (69.5)	1.00	
	A/G	49 (21.4)	65 (29.5)	1.52 (0.99–2.33)	0.060
	G/G	5 (2.2)	2 (0.9)	0.46 (0.09–2.39)	0.350
	A	399 (87.1)	371 (84.3)	1.00	
	G	59 (12.9)	69 (15.7)	1.26 (0.86–1.83)	0.230

### Comparison of rs35767 (G/A) polymorphism of IGF-1 gene between the T2DM group and the control group

The rs35767 genotype (GG, GA, AA), alleles (G, A), dominant model (GA + AA, GG) and recessive model (AA, GG + GA) of *IGF-1* gene in the T2DM and the control groups were also compared. The results showed that the frequency of dominant model GA + AA in T2DM group was significantly higher than that in control group (*P* < 0.05) (Table 6). GG of rs35767 of *IGF-1* gene indicated a lower risk of T2DM *(OR = *0.665, 95% CI = 0.458–0.965). The frequency of AA in the recessive model of the T2DM group was significantly higher than that of the control group (*P* < 0.05) (Table 6). Type GG + GA of rs35767 of *IGF-1* gene suggested a lower risk of T2DM (OR = 0.486, 95% CI = 0.247–0.957). There results suggest that rs35767 is associated with the occurrence of T2DM.

**Table 6:** Genetic model of the IGF-1 gene rs35767 locus in T2DM group and control group

SNP	Genotype/allele	Control group *n* (%)	T2DM group *n* (%)	OR (95%CI)	*P* value
Dominant model	AA + GA	109 (47.6)	127 (57.7)	1.00	
	GG	120 (52.4)	93 (42.3)	0.665 (0.458–0.965)	0.032
Recessive model	AA	14 (6.1)	26 (11.8)	1.00	
	GG + GA	215 (93.9)	194 (88.2)	0.486 (0.247–0.957)	0.034

### Interaction analysis of IGF-1 gene polymorphism

**Fig. 2:**
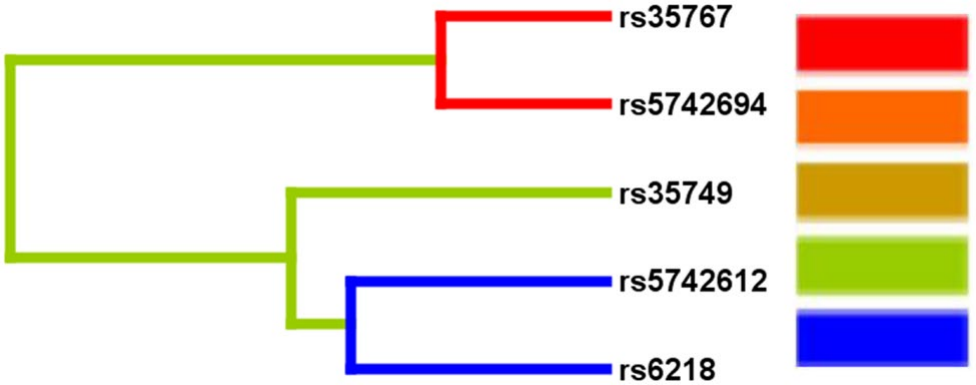
Tree diagram of interaction between SNP sites. Red and orange indicate that the interaction effect is synergistic, and the intensity of red is stronger than orange; blue and green indicate that the interaction effect is antagonistic, and the intensity of blue is stronger than green. It can be seen from the figure that the interaction between rs35767 and rs5742694 is highly synergistic

**Fig. 3:**
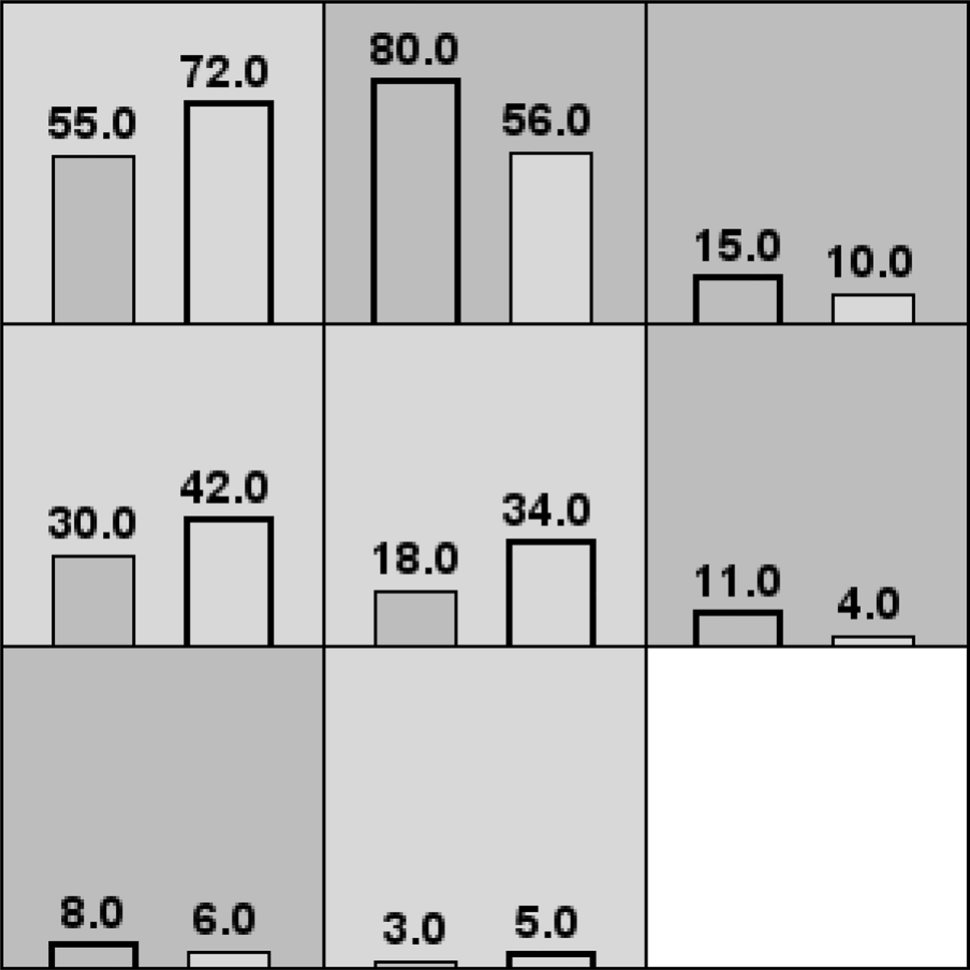
Distribution of high-risk and low-risk genotypes of interaction between rs35767 and rs5742694. High-risk combinations are represented by dark gray squares, while low-risk combinations are represented by light gray squares

The interaction of 5 SNP loci was analyzed by the GMDR method and 5 gene interaction models were generated. Among them, the two-factor model, which contained rs35767 and rs5742694, was the optimal gene interaction model with statistical difference (*P* < 0.05) and a large cross-validation consistency (10/10). We found that the interaction between rs35767 and rs5742694 was highly synergistic (Fig. 2). Furthermore, the combination of GG/CC, GA/AA, AA/AA, and AA/AC genotype of rs35767 and rs5742694 was in the high-risk group, whereas the combination of GG/AA, GG/AC, GA/AC and GA/CC genotype was in the low-risk group (Fig. 3). The risk of developing T2DM in the high-risk group was 2.16 times of that in the low-risk group (OR = 2.165, 95% CI = 1.478–3.171, Table 7).

**Table 7:** The best model of type 2 diabetes-related gene interaction

Model	Sample accuracy	Cross-validation consistency	*P* value
rs35767	0.4925	7/10	0.8281
rs35767 × rs5742694	0.5863	10/10	0.0107
rs35767 × rs5742694 × rs6218	0.5445	9/10	0.1719
rs35749 × rs35767 × rs5742694 × rs6218	0.5200	10/10	0.3770
rs35749 × rs35767 × rs5742612 × rs5742694 × rs6218	0.5193	10/10	0.3770

## Discussion

The participants (220 T2DM patients and 229 healthy controls) in this study were recruited from the Uyghur population in Urumqi, Xinjiang, China. The Uyghur residents in Urumqi are mainly from the northern and southern regions of Xinjiang. They are relatively less affected by the Han immigrants, and they have maintained a traditional way of life for generations, with their own language, religious beliefs and marriage custom (Black et al. 2006). Therefore, the Uyghur ethnic group is an ideal population for evaluation of genetic susceptibility and environmental factors. T2DM is a disease caused by genetic and environmental factors (Li et al. 2020). Susceptibility to T2DM varies among different ethnic groups. Studying the occurrence and development of T2DM in different ethnic groups is helpful to comprehensively understand the mechanism of this disease. However, there are relatively few studies on the genetic factors of T2DM in Uyghur population.

This study found statistical differences between the levels of TG, TC, HDL, LDL, BUN, and Cr between the T2DM group and the control group. Cohort studies conducted in China, South Korea, the United States and Japan also show that TG is associated with hyperuricemia and diabetes (McAdams-DeMarco et al. 2013; Qiu et al. 2013; Ryu et al. 2012). In T2DM patients, elevated TG, decreased HDL cholesterol, and increased LDL cholesterol are commonly seen (Blanco-Rojo et al. 2017; Zheng et al. 2020). These dyslipidemias may constitute the lipid profile of atherosclerosis and serve as risk factors for T2DM related cardiovascular disease (Howard et al. 2000; Ishikawa et al. 2015; Ye et al. 2019). Therefore, the regulation of dyslipidemias is an effective measure to control T2DM. In addition, it is reported that lower serum Cr level could increase the risk of T2DM (Larsen et al. 2016). The lifestyle, dietary habits and physical characteristics of the Uyghurs living in Xinjiang are somewhat different from those of the Han. It is shown that the Uyghurs have a high incidence of metabolic diseases (Dong et al. 2017). The Uyghurs’ diet is mainly composed pasta, beef and mutton. The intake of fats and dairy foods is significantly higher than the recommended amount in the Chinese Nutritional Dietary Guidelines; whereas the intake of vegetables, beans and fishery products is relatively low, resulting in high total calorie dietary structure and an increase in the overall overweight percentage of Uyghurs (Wang et al. 2013).

A number of studies have shown that excessive fat intake can cause insulin resistance (Kirsten et al. 2017; Bisschop et al. 2001; Wu et al. 2017). Wu et al. (2017) proposed that pancreatic β-cell dysfunction was a key factor in the development of T2DM. The increase in cholesterol levels in β-cells caused by the disorder of cholesterol metabolism, the hindrance of glycolysis, the increase of pancreatic β-cell apoptosis, and the decrease of insulin secretion were considered to be new mechanisms of β-cell dysfunction. Long-term living in an environment that easily leads to insulin resistance can also easily cause mutations in related genes. IGF-1 is a multifunctional cell growth regulator (Orru et al. 2017). It can be used as an intermediate in the process of insulin deficiency and hyperinsulinemia (Frystyk et al. 2002). Clemmons et al. found that the lack of IGF-1 could lead to more severe insulin resistance (Clemmons 2004). Low levels of IGF-1 can predict impaired glucose tolerance, T2DM, and cardiovascular disease (Dunger et al. 2003). It has been found that compared with other ethnic groups, rs35767 in IGF-1 may be the potential susceptible sites of T2DM in Uygur population (Song et al. 2015a, b). The results of this study also showed that the differences in genotype, allele frequency, dominant and recessive models of IGF-1 gene rs35767 locus between the T2DM group and the control group were statistically significant. Previous study has reported that the effects of the rs35767 polymorphism of *IGF1* on fasting insulin were mediated by decreased insulin sensitivity or impaired insulin (Mannino et al. 2013). The finding that the G allele of rs35767 (*IGF1*) is associated with fasting insulin and HOMA-IR in the European population (Dupuis et al 2010) and Han Chinese (Hu et al. 2010) is new.

We used G as the comparison group, and found that the A allele at rs35767 was associated with T2DM. Other study also revealed that the G allele at rs35767 was associated with reduced fasting insulin levels and insulin resistance (Hu et al. 2010). However, a meta-analysis of 76,558 non-diabetic populations of European descent found that the G allele at IGF1 rs35767 was associated with increased fasting insulin levels and insulin resistance (HOMA-IR) (Dupuis et al. 2010). The inconsistent results may be due to the influence of the disease microenvironment, the genotype distribution of different study populations or sample sizes. However, this study is consistent with the report by Manshu et al. (2015), which showed a significant difference in rs35767 (*IGF1*) G allele frequency between Uygur T2DM patients and control group. Additionally, compared with the control group, the frequency of GG genotype was lower in T2DM patients. These may be related to the dietary habits, living environment, and lifestyle of the Uyghur population discussed earlier. For example, blood glucose metabolism may affect the susceptibility of Uyghurs to diabetes (Zhang 2018). Moreover, the custom of internal marriage of the Uyghurs may also be a reason for the polymorphism of genetic susceptibility (Mamet et al. 2005; Wang et al. 2003).

In 2007, Lou et al. proposed GMDR, a method that can control the interference caused by covariates and improve the prediction accuracy (Lou et al. 2007). To study the interaction of diabetes-related genes, we analyzed the interaction of 5 SNP loci with the GMDR method. The results found that the two-factor model was statistically significant, and the cross-validation was consistent (10/10). The two-factor model contained rs35767 locus and rs5742694 locus, showing a high degree of synergy. Each G allele in rs35767 has a resistance of HOMA-IR (Hong et al. 2014). Similarly, Mannino et al. also suggested that subjects carrying with the GG genotype of rs35767 showed lower insulin sensitivity and IGF-1 concentration compared with those carrying the A allele (Mannino et al. 2013). The parameters of the rs5742694 polymorphism were reported for the first time as carriers of the TT genotype showing higher insulin sensitivity and lower insulin secretion (Willems et al. 2016). However, no significant correlation was found between the rs5742694 polymorphism and IGF-1 level in serum.

In conclusion, the rs35767 and rs5742694 in *IGF-1* may be potential susceptibility sites for T2DM in Uygur population. We believe that these findings will provide reference for personalized treatment of T2DM in the future.

## Data Availability

The data are available from the corresponding author on reasonable request.
